# Premature ovarian insufficiency: a review on the role of tobacco smoke, its clinical harm, and treatment

**DOI:** 10.1186/s13048-023-01330-y

**Published:** 2024-01-09

**Authors:** Jinghan Cui, Ying Wang

**Affiliations:** Department of Obstetrics and Gynecology, Shengjing Hospital, China Medical University, Shenyang, 110004 China

**Keywords:** Premature ovarian insufficiency, Tobacco smoke, Oocyte, Granulosa cells, Apoptosis, Oxidative stress

## Abstract

Premature ovarian insufficiency (POI) is a condition in which the quantity of follicles and the quality of oocytes gradually decrease. This results in an estrogen secretion disorder and abnormal follicle development, which can lead to related diseases, early onset of menopause, sexual dysfunction, and an increased risk of cardiovascular issues, osteoporosis, and depression, among others. This disease significantly impacts the physical and mental health and overall quality of life of affected women. Factors such as genetic abnormalities, oophorectomy, radiotherapy for malignancy, idiopathic conditions, and an unhealthy lifestyle, including smoking, can accelerate the depletion of the follicular pool and the onset of menopause. Extensive research has been conducted on the detrimental effects of tobacco smoke on the ovaries. This article aims to review the advancements in understanding the impact of tobacco smoke on POI, both in vivo and in vitro. Furthermore, we explore the potential adverse effects of common toxicants found in tobacco smoke, such as polycyclic aromatic hydrocarbons (PAHs), heavy metals like cadmium, alkaloids like nicotine and its major metabolite cotinine, benzo[a]pyrene, and aromatic amines. In addition to discussing the toxicants, this article also reviews the complications associated with POI and the current state of research and application of treatment methods. These findings will contribute to the development of more precise treatments for POI, offering theoretical support for enhancing the long-term quality of life for women affected by this condition.

**Supplementary Information:**

The online version contains supplementary material available at 10.1186/s13048-023-01330-y.

## Introduction

Premature ovarian insufficiency (POI) has emerged as a significant public health concern and a leading cause of fertility issues. As the quality of oocytes diminishes, and the quantity of follicles declines significantly, the female reproductive system undergoes an accelerated aging process, resulting in irregular menstruation and early onset of menopause. This, in turn, leads to ovarian dysfunction and infertility. Disorders in estrogen secretion and abnormal follicular development can further trigger various related health conditions, significantly impacting the overall quality of life for affected women. Genetic abnormalities, oophorectomy, radiotherapy for malignancies, idiopathic conditions, and unhealthy lifestyle choices such as smoking can expedite the depletion of the follicular pool and the onset of menopause [1].

The ovary, as a critical female reproductive organ, plays a pivotal role in ovulation and the secretion of sex hormones necessary for the subsequent growth, development, and maturation of follicles [2]. A woman is born with a fixed number of follicles in her ovaries, which gradually develop and mature, ultimately being ovulated during puberty [3]. This specific development process involves several key stages: gastrulation → ectodermal cell formation → primordial germ cells → germ cells → oogonia → primary oocytes → secondary oocytes → Graafian follicles → mature oocytes. The depletion of the follicle pool signals the onset of menopause, marking the conclusion of the follicle growth process. Diminished oocyte quality disrupts the intricate sequence of maturation, ovulation, fertilization, implantation, and early embryonic development, ultimately resulting in compromised reproductive health and infertility.

**Fig. 1:**
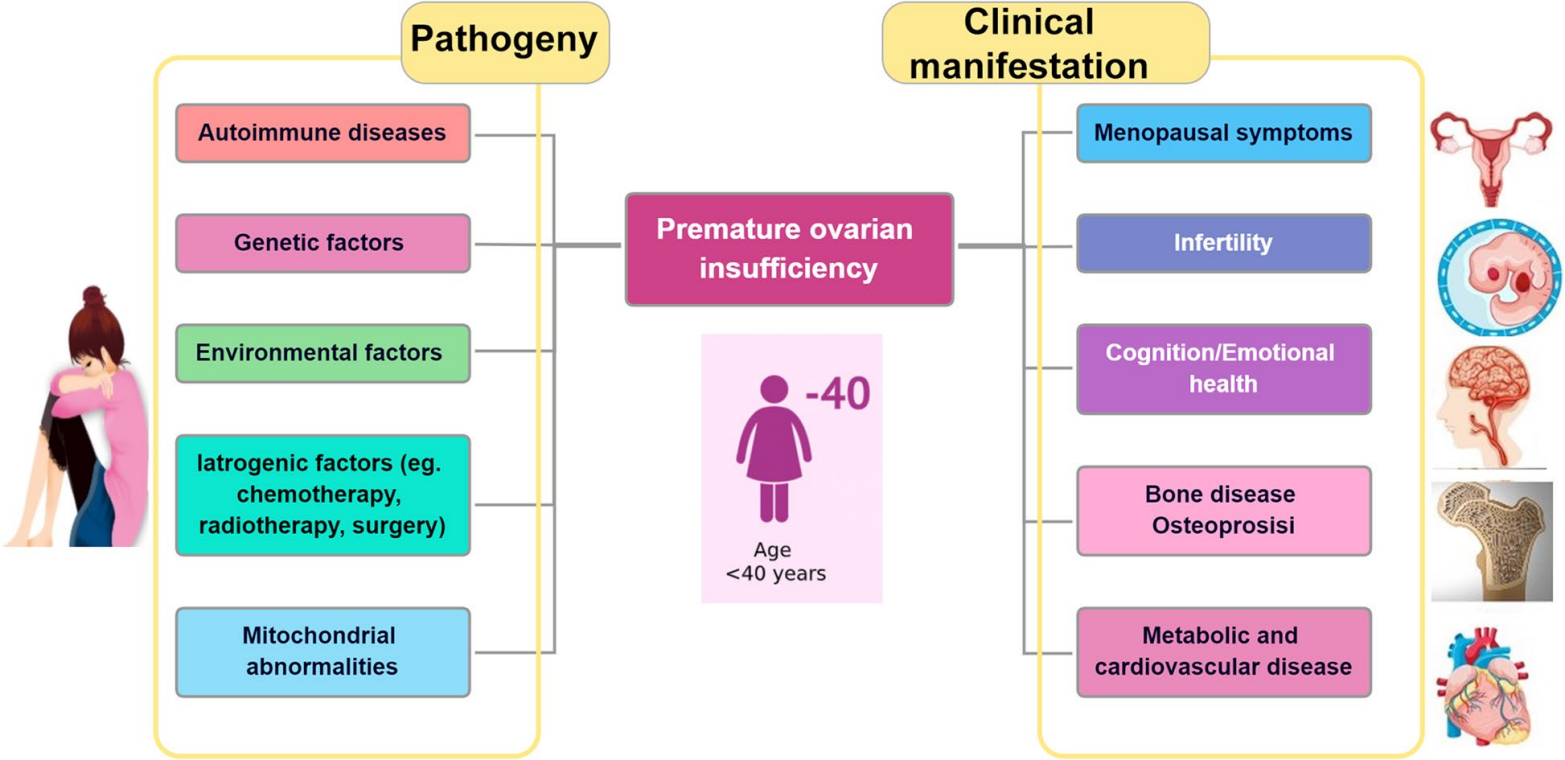
Pathogeny and clinical manifestation of premature ovarian insufficiency

According to the European Society of Human Reproduction and Embryology Guidelines (ESHRE) (European Society for Human et al. 2016), clinical symptoms of oligomenorrhea or amenorrhea persisting for at least 4 months, with intervals exceeding 4 weeks, and supported by multiple tests and examinations revealing a follicle-stimulating hormone (FSH) level greater than 25 IU/L, can define the current presence of POI. The prevalence of POI in females under 40 years of age is approximately 1% (European Society for Human et al. 2016). However, the causes and pathogenesis of POI remain unclear. The progression of POI can be categorized into four stages: the normal stage, the latent stage, the abnormal biochemical stage, and the clinical development stage. Premature ovarian failure (POF) represents the final stage of POI. Ovarian insufficiency can manifest as secondary amenorrhea, menstrual irregularities, reduced fertility, and symptoms of estrogen deficiency (European Society for Human et al. 2016). POI not only impacts women's fertility, causing them to enter menopause prematurely or experience sexual dysfunction, but it also elevates the risk of cardiovascular problems, osteoporosis, depression, and other diseases affecting physical and mental health (see Fig. 1). Extending the female reproductive lifespan reduces the risk of bone injuries and type 2 diabetes but increases the incidence of hormone-sensitive malignancies.

POI is an idiopathic disorder that originates from various factors, including autoimmune diseases [4], hereditary factors [5], environmental factors [6], iatrogenic factors [7] such as chemotherapy, radiotherapy, and surgery, and mitochondrial abnormalities [8]. Currently, approximately a quarter of patients diagnosed with POI have a relatively clear cause. Compounds like phthalates, bisphenol A, pesticides, and tobacco have been found to have toxic effects on ovarian function, leading to increased follicular exhaustion and early menopause. These detrimental effects have been observed at various stages from fertilized egg development to adulthood, with different mechanisms operating at each stage. However, the etiology of more than 75% of POI cases remains undetermined [9]. Notably, smoking has adverse effects on female fertility and is a recognized risk factor for POI [10]. Factors and genes related to sex hormones and the menstrual cycle appear to exert a more pronounced influence on smoking habits and cessation in women compared to men, underscoring the importance of considering gender differences in providing tobacco dependence interventions [11]. This article primarily aims to review the potential pathways through which tobacco smoke, both active and passive exposure, can cause ovarian damage. Additionally, it will explore the complications associated with POI and propose treatment plans.

### Effects of smoking on ovarian function and research status (Fig. 2)

**Fig. 2:**
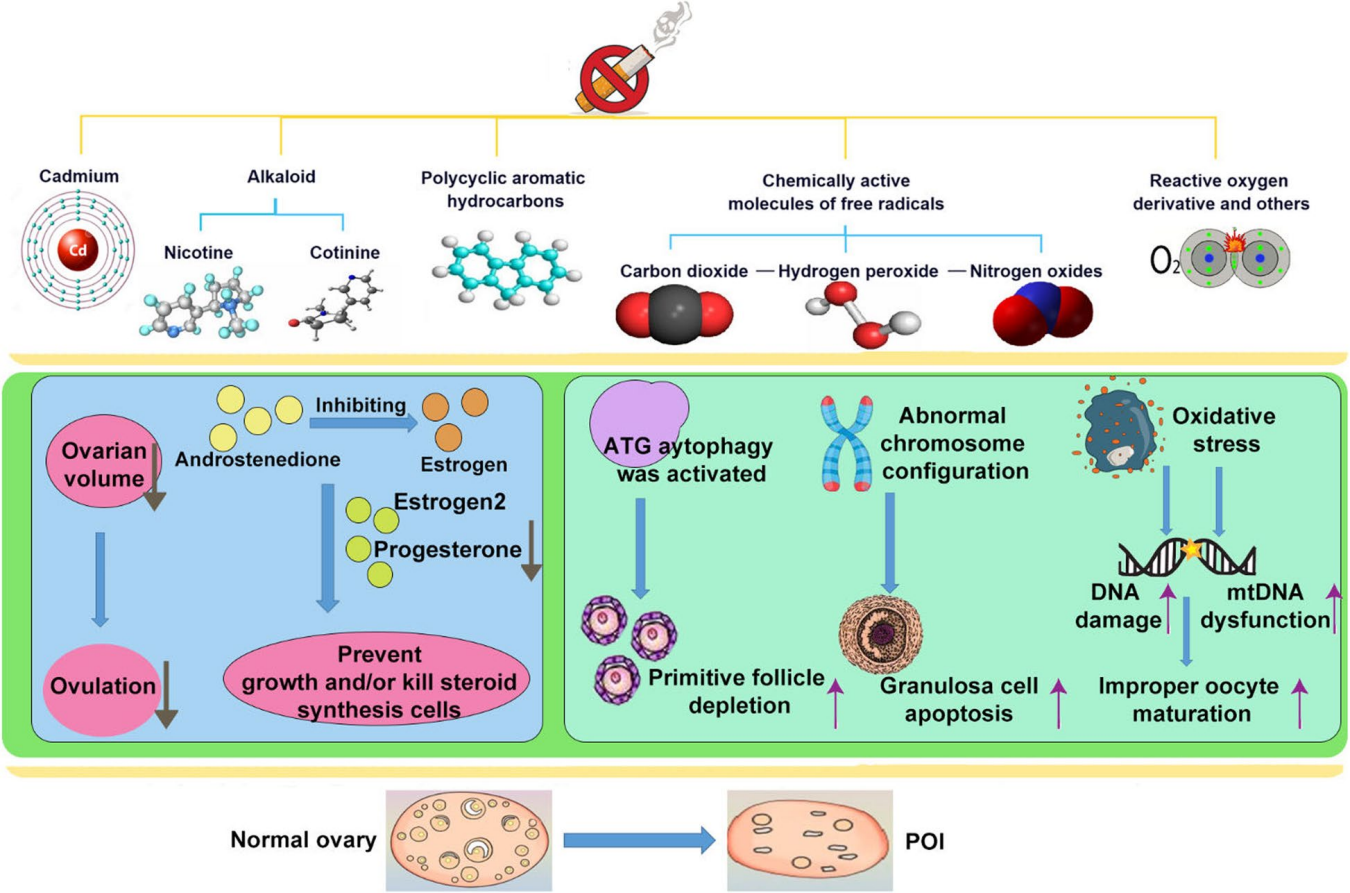
Main components of tobacco smoke and its damage to ovary

#### The effect of smoking on ovarian function

Environmental factors appear to be significant determinants of ovarian reserve and may play a role in premenopause during the prenatal period or adulthood [12]. Environmental toxins can exert deleterious effects by disrupting the process of communication and nutrient flow from granulosa cells to oocytes. Cigarette smoke contains nearly 5,000 toxic and harmful substances, which are believed to be mutagenic and carcinogenic. Smoking is a recognized risk factor for POI [13–15]. Tobacco smoke disrupts folliculogenesis and development, increasing apoptosis or autophagy, DNA damage, and abnormal connections between oocytes and granulosa cells that are associated with ovarian follicle death [16]. Human granulosa cells cultured in vitro showed reduced production of estradiol and progesterone when exposed to tobacco smoke [17]. Granulosa cells are the main components of ovarian somatic cells and play a vital role in follicular growth or atresia. Studies have indicated a significant increase in primordial follicle depletion and antral follicle apoptosis after exposure to tobacco smoke. There were indications of persistent oxidative stress in ovarian tissue and undamaged oocytes exposed to tobacco smoke, with marked increases in mitochondrial ROS and lipid peroxidation levels and CYP2E1 detoxification enzymes. This results in reduced fertilization potential and the appearance of whole oocyte dysfunction [18]. Women who smoke suffer from weakened ovarian function, disturbances in hormonal synthesis and metabolism, followed by reduced fertility, early menopause, and a decrease in ovarian reserve. In recent years, the global smoking rate has been declining, but the likelihood of smoking among women of childbearing age has been significantly increasing worldwide, with nearly 33.3% of this age group having a history of smoking [3].

Research has shown that various pollutants can impact all gametogenesis processes and may have adverse effects on ovarian function in women post-birth [19, 20]. It has been documented that exposure to cigarette smoke can lead to a reduction of approximately 20% in women's ovarian reserve (Zenzes 2000). Some women exposed to cigarette smoke may even experience premature menopause [21]. Moreover, a different set of studies has revealed that mothers who smoked during pregnancy had a significantly decreased quantity of oocytes and somatic cells in the ovaries of their female offspring [22], leading to reduced fertility and an earlier onset of menopause [23]. In a study utilizing a mouse model of chronic obstructive pulmonary disease induced by cigarette smoke, female mice exposed to direct nasal smoke showed signs of primordial follicle depletion, sinus follicle oocyte apoptosis, induced oxidative stress, and decreased follicular ovulation [18]. Smoking exposure has been linked to a reduction in the follicle reserve in ovaries and alterations in estrus status and serum hormone levels [16].

Previous research has demonstrated that women exposed to cigarette smoke experience a significant reduction in ovarian volume compared to women of the same age who were not exposed to cigarette smoke [24]. Upon microscopic examination, a pronounced decrease in the quantity of follicles was observed, primarily affecting primordial follicles, without a notable impact on primary, secondary, or antral follicles. Cigarette smoke contains various toxic substances, including PAHs, heavy metals such as cadmium, alkaloids like nicotine and its main metabolite cotinine, benzo[a]pyrene, and aromatic amines. Polycyclic hydrocarbons present in tobacco smoke may potentially harm ovarian germ cells, leading to follicular dysfunction and decreased blood estrogen levels [13, 25]. Increased oxidative stress, heightened apoptosis, alterations in the relationship between granulosa cells and oocytes, impaired oocyte nuclear function, and compromised luteal cell integrity may collectively contribute to disruptions in follicular development, possibly representing the mechanisms behind tobacco-induced damage to reproductive function [26]. In vitro studies have revealed that alkaloids found in tobacco, such as nicotine, cotinine, and anabacin, inhibit the conversion of androstenedione to estrogen, consequently leading to reduced estrogen levels in the bloodstream [27, 28]. Animal studies have indicated that smoking can impact the functioning of the hypothalamic-pituitary axis [29], leading to rapid hormonal imbalances release [13, 30]. Exposure to tobacco smoke does not induce apoptosis of Bcl-2/Bax-associated granules but activates the Atg autophagy pathway, ultimately resulting in follicular loss in mice. Smoking disrupts the menstrual cycle, reduces the efficacy of hormone replacement therapy, and increases the likelihood of experiencing side effects. Research suggests that the deleterious effects of smoking on ovarian function may be irreversible.

The literature suggests that increased smoking is associated with early menopause [31, 32], and that women who smoke enter menopause one year earlier than non-smoking women [13]. Genetically mediated increases in alcohol consumption and smoking are associated with early age at natural menopause (ANM). For each additional cigarette smoked per day, ANM was reduced by about 2.5 weeks [33]. The occurrence of premature menopause may be related to the amount of smoking, and the sooner women quit smoking, the more likely they are to avoid early menopause [34]. Some studies suggest that both passive and active smoking are associated with early menopause [35], while others report that only active smoking has this effect [35, 36]

### The mechanism of common toxins in tobacco smoke affecting ovarian development and function

#### The effect of cadmium on ovarian development and function and its possible mechanisms

Cadmium, a heavy metal commonly found in plastic products, soil, animals, and food such as meat and fruit, is considered a major environmental pollutant. However, the most common modes of exposure to cadmium are still believed to be from tobacco smoke pollution. Cadmium has a biological half-life as long as 15 to 30 years and is slowly excreted from the body [37]. It inhibits the production of steroid hormones, such as progesterone, which is essential for follicular growth [38, 39].

Different concentrations of cadmium can have a dual effect on steroidogenesis. Previous studies have detected cadmium in the follicular fluid and oocytes of smokers [40, 41]. When the concentration of cadmium in female follicular fluid reaches 6.73 ± 0.31 μg/L, it interferes with the growth and development of follicles, increases the number of follicular atresia, and can further lead to ovulation failure, implantation disorders, spontaneous abortion, and birth defects [42, 43]. mRNA level studies have shown that cadmium can cause ovarian development disorders in rats by downregulating the expression of the SCF/c-kit gene and its related microRNA factors [44].

In rodents, under the electron microscope, cadmium particles are deposited in the ovary, destroying the structure and function of the ovary. This damage affects the endoplasmic reticulum, mitochondria, Golgi apparatus, endometrium, and other cellular organs. It also degrades the corpus luteum and disrupts the arrangement of follicles and corpus luteum, increasing the number of atretic follicles [42, 45, 46].

#### The effect of benzo[a]pyrene on ovarian development and function and its possible mechanisms

Benzo[a]pyrene can be detected in human serum and follicular fluid. It is found at higher concentrations in the follicular fluid of women exposed to tobacco smoke, with its levels being positively correlated with exposure [47]. Furthermore, the concentration of benzo[a]pyrene in the lung tissue of smokers is approximately twice as high as that in non-smokers [48]. The study revealed that the concentration of benzo[a]pyrene (B[a]P) was negatively correlated with the level of E2 in the medium. A decrease in E2 may result in insufficient follicular growth and development. E2 primarily exerts its effects through estrogen receptor alpha (ERa) or beta (ERb). Granulosa cells predominantly express ERb, a receptor that regulates granulosa cell growth and the effects of FSH. Animal experiments demonstrated that when rats were exposed to high levels of B[a]P, both E2 secretion and cumulus cell proliferation significantly decreased [47].

In rodent studies, benzo[a]pyrene (B[a]P) and other PAHs were shown to reduce external follicular growth [47], while in vivo exposure resulted in the loss of primordial follicles through apoptosis induction. In utero exposure to B[a]P led to a decreased quantity of primordial follicles and infertility in mice [49]. Earlier studies have indicated that B[a]P damaged DNA in human granulosa cells. However, recent research has shown that B[a]P is more concentrated in the follicular fluid compared to serum, suggesting that it may be preferentially accumulated in the ovary, leading to ovarian toxicity and selective destruction of preantral follicles, including primitive and primary follicles [47].

#### The effect of alkaloids (nicotine and its major metabolite cotinine) on ovarian development and function and its possible mechanisms

The content of alkaloids in tobacco smoke is relatively high (2010), with nicotine being the primary alkaloid, along with its main metabolites, nornicotine and cotinine. Nicotine exhibits a certain degree of anti-estrogenic effects, further disrupting the balance of reproductive and hormonal systems, and reducing the likelihood of successful pregnancy in both healthy women and those undergoing assisted reproduction [50]. Smoking can exacerbate the symptoms of premenstrual syndrome due to nicotine's impact on neural circuits, which increases sensitivity to environmental stressors [51]. Nicotine in cigarettes activates the sympathetic adrenal system, leading to increased synthesis and release of norepinephrine and adrenaline. These effects can result in an elevated heart rate, increased blood glucose levels, weight loss, and enhanced stimulation of the hypothalamic–pituitary–adrenal (HPA) and hypothalamic-pituitary-thyroid (HPT) axes, ultimately leading to elevated plasma cortisol and thyroid hormone concentrations [52]. Cotinine, with a biological half-life estimated to be approximately 16–19 h [53], serves as a valuable marker of cigarette smoke exposure due to its extended half-life [54]. Potential targets for these toxins include the nervous system, kidneys, liver, heart, and reproductive organs. Estrogen and progesterone, both essential for follicle growth, oocyte maturation, and ovulation, are steroid hormones that require synthesis from cholesterol. Nicotine can influence basal luteosterol production in human granulosa cells but has no significant effect on hCG-promoted progesterone release. Studies have indicated that nicotine and its metabolites cause human luteal insufficiency by regulating the prostaglandin (PG) system, severely inhibiting progesterone release. This inhibition of progesterone by tobacco smoke alkaloids appears to be associated with growth arrest and/or apoptosis of steroidogenic cells [55].

When nicotine was administered to oocytes at low levels (< 0.5 mM), there was no significant influence on their maturation. However, exposure to higher concentrations (5 mM) led to significant changes in subsequent meiosis, resulting in abnormal chromosomal configurations [56]. Animals exposed to high doses of nicotine exhibited an increased proportion of granulosa cells undergoing apoptosis in the follicles, further inhibiting follicle growth. Follicle growth is a crucial process in ovulation and fertilization, and nicotine-induced apoptosis of granulosa cells may be a significant mechanism in the context of cigarette smoke and fertility-related diseases.

The ovary expresses transcripts from different subunits of nAChR, and studies have shown that the expression levels of some transcripts are significantly altered after exposure to nicotine. Various cell types of different origins and functions express nicotine receptors, including nAChR, within the granulosa cells of the adult rat ovary. Some cells synthesize and release acetylcholine [57, 58]. The pharmacological properties of nAChRs promote a highly tissue-specific response to circulating levels of nicotine ligands. Through their interaction with nAChRs, several ligands can modulate the activity of specific nAChR isoforms, influencing biological functions such as cell proliferation, apoptosis, migration, and signal transduction [58].

#### The effects of polycyclic aromatic hydrocarbons on ovarian development and function and their possible mechanisms

PAHs in tobacco smoke are believed to exert significant toxic effects on the ovary. Studies have demonstrated that women who smoke exhibit lower pregnancy rates per menstrual cycle and experience early menopause [13]. In experimental research, various methods of exposing adult mice to various PAHs have induced ovarian tumorigenesis [59–61]. Epidemiological studies have revealed an elevated risk of developing epithelial ovarian cancer in women who smoke [62, 63].

PAHs are highly mutagenic and can lead to conditions such as mammalian primordial folliculopenia, follicular atresia, and overall ovarian toxicity. Exposure of ovaries to PAHs in vitro has been shown to increase the expression of Bcl-2, while the protein expression of pro-apoptotic markers such as Bax and active caspase 3 remains unchanged, with no significant alteration in the level of apoptosis. One specific PAH, 9,10-dimethylbenzo(a)anthracene-3,4-dihydrodiol (DMMA-DHD), is known to activate the aryl hydrocarbon receptor (AHR) present in human ovarian and reproductive cells [64]. This activation further induces fetal germ cell apoptosis by directly stimulating Bax expression [65]. Early exposure of fetal ovaries to PAHs during pregnancy has been associated with reduced ovarian germ cell proliferation [66]. Tobacco smoke accelerates oocyte atresia and impairs oocyte quality, which are the two primary factors contributing to the shortened female reproductive lifespan.

#### The effects of other harmful substances on ovarian development and function and their possible mechanisms

Various chemically active molecules, such as carbon dioxide, hydrogen peroxide, and nitrogen oxides, containing reactive oxygen derivatives and free radicals, are present in tobacco smoke. Apoptosis in follicle cells significantly increased due to oxidative stress, and a glutathione culture demonstrated that antioxidants can prevent excessive cell death [3]. When examining human ovarian cells, the primary toxic effects of smoking were observed to be oxidative stress and DNA damage in granulosa cells and cumulus cells of oocytes. This damage further resulted in impaired ovarian function, including improper maturation of oocytes, compromised binding of gonadotropins to their receptors, and an increase in the thickness of the transparent zone, which could reduce fertilization ability [67–70].

Both enzymatic and non-enzymatic antioxidants were found to be reduced in smokers [71]. The study also revealed that the antioxidant capacity of follicular fluid is generally low. High levels of nitric oxide (NO) produced by oocytes or follicular somatic cells play a crucial role in oocyte maturation and physiological processes. A decrease in NO levels can lead to high concentrations of free radicals causing cellular damage [72, 73]. Melatonin, known as N-acetyl-5-methoxytryptamine, can be derived from various tissues, such as the nervous system, immune system, and reproductive system [74, 75]. It serves as both a neurohormone and an antioxidant molecule. Melatonin and its metabolites exert their effects by scavenging reactive oxygen and reactive nitrogen species (RNS) [76],El-Sokkary et al. 2006). Treatment with different concentrations of melatonin in smokers can lead to a significant decrease in the apoptotic index of follicle cells, while simultaneously increasing tissue antioxidant activity [77].

Previous studies have identified more than 50 mutations in multiple genes, including LHCGR, FSHR, NR5A1 [78], NOBOX [79], FIGLA, BMP15 [80], FOXL2, STAG3, NANOS3 [81], as contributing factors in idiopathic POIs. The pathogenic factors of POI [82–84] involve the interplay of genetic changes and environmental factors, and the precise pathogenesis remains unclear.

### Complications of POI (Table S1)

Impaired fertility represents the most distressing consequence of POI, imposing a significant psychological burden on young women. The presence of impaired fertility and the fear of premature aging are linked to a loss of emotional control, diminished self-esteem, social anxiety, and an increased incidence of depressive symptoms [85]. Currently, there are no clearly effective interventions to enhance residual ovarian function, with the primary approach being the use of donated eggs to improve the conception rate among women with POI (van Kasteren and Schoemaker 1999; [86]. Nevertheless, infertility associated with POI is not an absolute condition. In about 25% of patients diagnosed with POI, ovulation may still occur, potentially leading to pregnancy and childbirth. Furthermore, it is worth noting that women with idiopathic POI who conceive naturally do not exhibit a higher incidence of obstetric complications or neonatal risks when compared to the general population [86].

Cardiovascular disease and stroke stand out as the leading causes of reduced life expectancy among untreated POI patients compared to age-matched controls (Archer 2009; [86, 87]. This primary cause may be attributed to the diminished vascular endothelial function observed in POI patients, which in turn contributes to the development of atherosclerosis [88]. Estrogen proves to be advantageous for cholesterol metabolism, capable of reducing the incidence of atherosclerosis, and alleviating coronary artery constriction through catecholamine regulation [89]. Furthermore, endothelial function demonstrated significant improvement following estrogen therapy [90]. A consistent body of research supports that, in patients with POI, the benefits of estrogen therapy far outweigh any associated risks [91]. Women who experience premature menopause due to bilateral oophorectomy for medical reasons exhibit a substantial cognitive decline [92, 93], and the earlier the surgical intervention, the more rapid the cognitive decline tends to be [94]. An increased formation of long-term neuritic plaques amplifies the risk of Alzheimer's disease neuropathy [92, 93]. It should be noted that while supplemental estrogen therapy leads to an improvement in the degree of cognitive decline, it does not reverse the neuropathology associated with Alzheimer's disease [92, 93].

Bone mineral density (BMD) experiences a significant decrease in patients with POI [88, 95]. Lifestyle modifications, such as engaging in weight-bearing exercise, increasing calcium intake, supplementing with vitamin D, and refraining from smoking, promote overall bone health [86]. It is currently recommended to guide women with POI to consider estrogen replacement therapy prior to natural menopause, with the aim of reducing the risk of chronic diseases such as cardiovascular disease, osteoporosis, and neurocognitive impairment [86, 87].

### Treatment of POI and prospects for treatment

Hormone replacement therapy (HRT), which is considered a physiological replacement for estrogen, is currently a common treatment for POI. However, it does not fully restore ovarian function. Therefore, there are various new methods under review for addressing this issue. These methods include stem cell activation, in vitro activation (IVA), mitochondrial activation, exosome therapy, biomaterial strategies, and intraovarian perfusion of platelet-rich plasma (PRP).

Stem cell activation can occur through the release of paracrine bioactive molecules, such as cytokines, regulatory factors, growth factors, and signal peptides. These molecules can have a positive impact on neighboring cells, improving ovarian function by exerting anti-inflammatory, anti-apoptotic, anti-angiogenic, anti-fibrotic, and immune-regulating functions [96]. In stem cell research, the primary focus has been on various types of stem cells, including bone marrow stem cells (BMSCs) [97–100], umbilical cord mesenchymal stem cells (UC-MSCs) [101, 102], menstrual blood-derived mesenchymal stem cells (MenSCs) [103, 104], human amniotic fluid stem cells (AFSCs) [105, 106], placenta-derived mesenchymal stem cells (PMSCs) [107–110], amniotic mesenchymal stem cells (AMSCs) [111, 112], and adipose-derived stem cells (ADSCs) [113, 114]. In addition, some researchers have proposed that induced oogonial stem celled (iOSC) may provide a new direction for the treatment of POI [115] (Table 1).

**Table 1:** Summary of stem cells for POI therapy

Type	Author	Year	Mechanisms	Outcome
Bone marrow stem cells (BMSCs)	S. Abd-Allah et al	2013	The secretion of vascular endothelial growth factor (VEGF) increased	The level of FSH decreased, the number of follicles and estrogen levels increased
	J. Guo et al	2013	1. Up regulation of c-myc proto oncogene mRNA 2. Down regulation of bcl 2 related X protein (Bax) and cyclin dependent kinase inhibitor 1a (p21)	Inhibition of granulosa cell apoptosis
	Y. Zhang et al	2022	Exosomal mir-644-5p and mir-144-5p regulate p53 and target PTEN	Inhibition of granulosa cell apoptosis
	X. Fu et al	2017	MiRNA down regulates programmed cell death protein 4 (PDCD 4) and PTEN	Repair ovarian structure and function
Umbilical cord mesenchymal stem cells (UC MSCs)	L. Cui et al	2020	Transforming growth factor- β/ Smad3 signaling pathway regulates ovarian stromal cell differentiation	Inhibit the occurrence and development of ovarian fibrosis in POI rats
	C. Ding et al	2020	Exosomal mirna-17-5p regulate sirt7	Improve ovarian function
Menstrual blood derived mesenchymal stem cells (MenSCs)	Z. Yan et al	2019	Increase the secretion of fibroblast growth factor 2	Avoid granulosa cell apoptosis
Human amniotic fluid stem cells (AFSCs)	X. Yu et al	2014	Potential for differentiation into primordial follicle oocytes	Improve ovarian function
	G. Xiao et al	2016	Exosomes mir-10a and miR-146a can inhibit follicular atresia	Improve the survival rate of granulosa cells
Placental derived mesenchymal stem cells (PMSCs)	H. Zhang et al	2018	Increased serum AMH and estrogen secretion	Inhibit follicular atresia and granulosa cells apoptosis
	J. Seok et al	2020	Up regulation of antioxidant factors	Inhibit granulosa cells apoptosis
	N. Yin et al	2018	Regulation of the ratio between Th17 / Treg and Th17 / T C17 cells through PI3K / Akt signaling pathway	Inhibit granulosa cells apoptosis
	H. Li et al	2019	Inhibition of endoplasmic reticulum stress inositol requires enzyme 1 signaling pathway	Inhibit granulosa cells apoptosis
Amniotic mesenchymal stem cells (AMSCs)	L. Ling et al	2017	Reduce the expression of inflammatory cytokines	Inhibit the occurrence and development of ovarian inflammation in POI rats
	C. Ding et al	2020	Exosomal mir320a inhibit ROS production by regulating sirtuin4	Alleviating POI
Adipose derived stem cells (ADSCs)	P. Terraciano et al	2014	Induce angiogenesis	Restore the number of follicles and repair the damage of ovary
	B. Huang et al	2018	Exosomes regulate the Smad pathway	Restore ovarian function in patients with POI
Induced oogonial stem celled (iOSC)	O. Celik et al	2020	Mesenchymal stem cells (MSCs) were cultured and isolated in vitro, and OSC-specific genes were transferred into MSCs to produce iOSC	Intraovarian transplantation of iOSCs can produce oocyte like cells with haploid chromosomes

*Abbreviation*: *FSH* Follicle stimulating hormone, *AMH* Anti-Müllerian hormone, *POI* Primary ovarian insufficiency, *ROS* Reactive oxygen species

The tumorigenicity, immunogenicity, and heterogeneity of stem cells currently limit their applications, with a particular emphasis on addressing tumorigenicity. One emerging approach under investigation involves the intraovarian infusion of PRP as a novel treatment method for POI. In this method, activated platelets stimulate angiogenesis, control inflammation, and promote anabolism by releasing a multitude of hormones and growth factors, thereby facilitating tissue healing and regeneration [116]. The potential risks associated with this approach primarily include intense cellular proliferative events, which may pose a risk of malignancy, as well as the potential for infection and unknown adverse effects on the embryo. Most current studies on the treatment of POI are based on animal models. However, significant differences exist between humans and animals, underscoring the need for more precise experiments in the future to enhance the safety and efficacy of these new technologies in human applications.

## Conclusion

In conclusion, POI can manifest through various mechanisms, including reduced peak follicle quantity, accelerated follicle depletion due to apoptosis, or follicle dysfunction [117]. This review offers a comprehensive understanding of the impact of tobacco smoke on POI, particularly the effects and potential mechanisms of various harmful substances such as PAHs, heavy metals (cadmium), alkaloids (nicotine and its primary metabolite, cotinine), and benzo[a]pyrene on ovarian development. Furthermore, it consolidates the range of complications associated with POI and highlights current treatment methods, thereby providing valuable insights for further exploration into the prevention and management of this condition.

### Supplementary Information

**Additional file 1:** **Table 1.** Complications of POI.
