# Supplementary material for: Premature ovarian insufficiency: a review on the role of tobacco smoke, its clinical harm, and treatment
Source: J Ovarian Res. 2024 Jan 9;17:8. doi: 10.1186/s13048-023-01330-y (PMC10775475; doi:10.1186/s13048-023-01330-y)
Supplement: Supplementary file 1 — Additional file 1: Table 1. Complications of POI. [file 13048_2023_1330_MOESM1_ESM.docx]

Table 1 Complications of POI

| Author | Year | Complications | Main mechanism | Current treatment options |
| --- | --- | --- | --- | --- |
| P. Schmidt et al | 2006 | Impaired fertility. | Decreased ovulation. | Donor Eggs Increase Conception Rates. |
| L. Webber et al | 2016 | Heavy psychological burden. | Impaired ability and fear of premature aging. | No effective means available at this time. |
| S. Sullivan et al | 2016 | Cardiovascular disease and stroke. | Decreased vascular endothelial function. | Estrogen replacement therapy before natural menopause. |
| V. Popat et al | 2009 | The risk of fracture is significantly increased. | Bone mineral density (BMD) decreased significantly. | Make lifestyle changes, increase calcium and vitamin D intake and avoid smoking. |
| P. Sarrel et al | 2016 | Cognitive decline. | The formation of neuritis plaque increased. | Estrogen replacement therapy before natural menopause. |
